# A data collection of UML diagrams in PlantUML notation mined from open-source software

**DOI:** 10.1016/j.dib.2026.113156

**Published:** 2026-08-08

**Authors:** Volodymyr Polishchuk, Vladyslava Skidan

**Affiliations:** Kyiv National University of Technologies and Design, Mala Shyianovska (Nemyrovycha-Danchenka) Street, 2, Kyiv, 01011, Ukraine

**Keywords:** Unified modeling language, Diagram-as-code, Software repository mining, Diagram classification, Large language models, Empirical software engineering, Model-driven engineering

## Abstract

Unified Modeling Language (UML) diagrams are widely used in software engineering education and practice. However, research on real-world UML usage has been constrained by the limited availability of large-scale, openly accessible, and structurally annotated diagram collections, with existing resources differing in size, format, and the absence of per-diagram type labels.

To address this gap, this data article presents a collection of 143,427 UML diagrams in PlantUML notation, mined from open-source software repositories indexed by the World of Code (WoC) infrastructure. The data pipeline identifies PlantUML files by file extension across World of Code, validates the presence of PlantUML content markers, and compiles the validated files with PlantUML to confirm syntactic correctness.

Each diagram is then assigned to one of nine standard UML types using a large language model (LLM)-based classifier, and processed by a parser-based tool that extracts per-type counts of 57 structural element kinds and 28 connection kinds. The collection comprises class (51.6%), sequence (24.6%), activity (6.8%), component (5.7%), use case (5.0%), state (3.0%), object (1.7%), deployment (1.4%), and timing (0.1%) diagrams. Each diagram is released as both PlantUML source code and a rendered Portable Network Graphics (PNG) image, accompanied by metadata that records the assigned diagram type, the structural element and connection counts, textual size metrics, and source repository attribution.

The collection is published on Zenodo. Anticipated reuse scenarios include empirical analyses of UML usage in open-source software, training and evaluation of artificial-intelligence models for diagram understanding and generation, benchmarking of diagram-to-code and code-to-diagram translation tools, and the development of educational resources for software engineering.

Specifications Table

**Table:** 

Subject	Computer Sciences
Specific subject area	Software repository mining; classification and structural analysis of Unified Modeling Language (UML) diagrams in open-source software
Type of data	Text (PlantUML source code, .puml); Image (rendered diagrams, .png); Table (JSON metadata with aggregate statistics and per-diagram classification)
Data collection	PlantUML files were identified by file extension (.puml, .pu, .plantuml, .wsd, .iuml, .uml) across the World of Code (WoC) version V (May 2023 release), deduplicated by SHA-1 content hash, and validated for the presence of @startuml/@enduml markers. Each candidate file was compiled with PlantUML version 1.2025.9 to confirm syntactic correctness, then classified into one of nine standard UML types by Claude Haiku 4.5 (Anthropic Message Batches API). Per-diagram element and connection counts were extracted with DiagramStatsExtractor, a custom Java tool that reuses PlantUML's parser.
Data source location	The PlantUML files were extracted from the World of Code (WoC) infrastructure (https://worldofcode.org/), version V, which indexes open-source git repositories from GitHub, GitLab, Bitbucket, and other public forges.
Data accessibility	Repository name: Zenodo Data identification number (DOI): 10.5281/zenodo.20537143 Direct URL to data: https://doi.org/10.5281/zenodo.20537143 License: CC BY 4.0 (Creative Commons Attribution 4.0 International) for the authors' contributions (metadata, validation records, and curation); each .puml source file and rendered .png image remains subject to its upstream repository license.
Related research article	None

## 1 Value of the Data

**Fig. 1:**
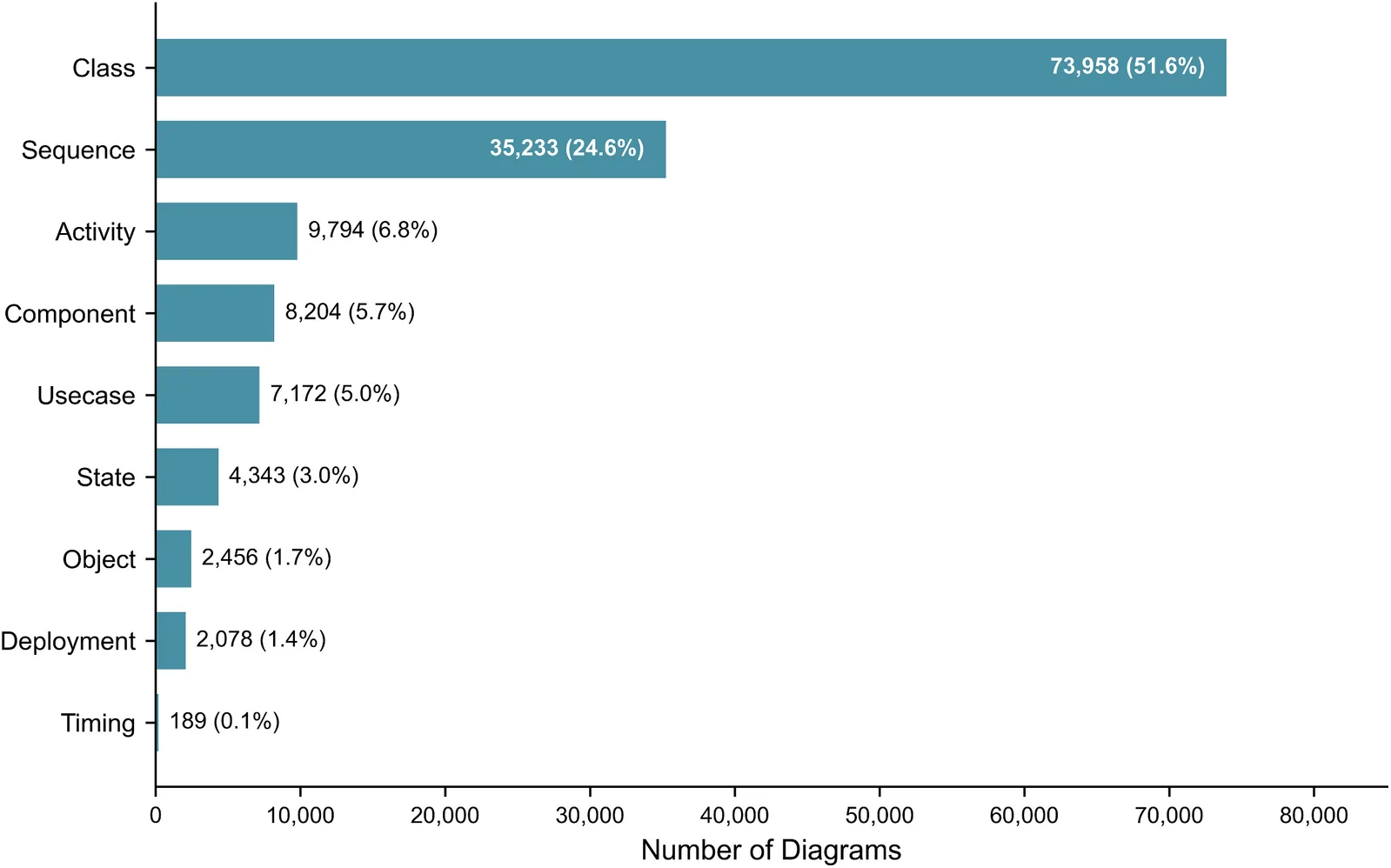
Distribution of UML diagram types in the dataset.
• JSON metadata provides per-diagram counts of typed structural elements and connections, together with each diagram's line count, supporting analyses of diagram complexity and structural patterns without requiring users to re-parse the source files. Example analyses include how complexity varies across the nine diagram types, which structural relationships recur most often, and how a diagram's structural size compares with its length in lines.
• Each diagram retains its blob-level provenance (original repository path and SHA-1 content hash), and the full extraction pipeline code is openly available, supporting reproducibility and reuse in research, model training, and educational materials.

• The dataset includes 143,427 UML diagrams in PlantUML notation, mined from open-source software repositories indexed by the World of Code infrastructure, and supports large-scale empirical analyses of UML usage in open-source projects, addressing questions such as how the popularity of each diagram type varies from one project to another, whether UML use is concentrated in a few projects or spread across many, and where in a repository diagrams are kept.
• Each diagram is released as both a compilable PlantUML source file and a rendered PNG image, and is labelled with one of nine standard UML types (class, sequence, activity, component, use case, state, object, deployment, timing), supporting text-based and image-based reuse for tasks such as diagram-to-code generation, code-to-diagram translation, automatic UML type classification, and visual question answering. Although the type distribution is unequal, with class and sequence diagrams together accounting for 76.2% of the dataset (Fig. 1), the per-diagram type labels support constructing type-balanced subsets.

## 2 Background

The Unified Modeling Language (UML) is a widely used notation in software engineering education and practice. Two lines of research depend on diagram corpora at scale: empirical analyses of UML usage in authentic software projects, and image-to-code generation, in which multimodal large language models are prompted to produce PlantUML source from rendered diagram images [1,2]. Both require diagrams drawn from real sources, annotated by type, and paired with their rendered images.

Prior open-source mining efforts have not met these requirements. The Lindholmen collection retrieved over 93,000 UML files from GitHub in image, XMI, and textual formats, but did not classify diagrams by type and did not include PlantUML [3,4]. A subsequent mining study identified PlantUML as the most prevalent textual UML format in open-source projects by file extension, but applied selection criteria (over 2000 commits, 100 stars, and 10 contributors) that yielded a corpus of 9875 files from 552 projects, and did not classify diagrams by type [5]. The World of Code (WoC) infrastructure now indexes open-source repositories across GitHub, GitLab, Bitbucket, and other public forges, making file-level extraction tractable across the open-source ecosystem [6], but has not been used to assemble a UML diagram corpus. The data described here comprise 143,427 PlantUML diagrams extracted from WoC version V, each labelled by diagram type, paired with its rendered PNG image, and accompanied by parser-based structural metrics.

## 3 Data description

The dataset [7] is published on Zenodo and comprises four data components: the PlantUML source files for all 143,427 diagrams, the corresponding rendered Portable Network Graphics (PNG) images (minimum resolution 512 by 512 pixels, RGB colour mode), a single JavaScript Object Notation (JSON) metadata file that records per-diagram type, structural metrics, and provenance, and a 270-diagram manually validated subset. The source files, images, and validation records are distributed as compressed archives that expand to directories of the same name. The image component contains 144,487 files, exceeding the 143,427 source files because some diagrams use the PlantUML newpage keyword, which produces multiple output pages from a single source file. Per-diagram content lines range from 1 to 31,784, with a median of 22 (mean 39.34, Q1 11, Q3 42).

Image widths range from 512 to 16,384 pixels, with a median of 1972 (mean 2703, Q1 1268, Q3 3207); heights span the same range, with a median of 1427 (mean 1869, Q1 863, Q3 2311). The 16,384-pixel cap is the renderer's bitmap limit, reached by 1587 images (1.1%) on at least one axis. Aspect ratios range from 0.03 to 32.0, with a median of 1.38. Users requiring a uniform input size for machine-learning models can apply aspect-ratio-preserving downscaling followed by padding to the target dimensions, sampling the pad colour from a corner pixel to match the diagram's page background.

Table 1 lists every file and directory in the dataset.

**Table 1:** Dataset file and directory listing.

File or directory	Description
LICENSE	Creative Commons Attribution 4.0 International (CC-BY-4.0) license text
README.md	Dataset description and usage notes
uml_metadata_enriched.json	Per-diagram metadata for all 143,427 diagrams
puml_files.zip	Archive of 143,427 PlantUML source files
puml_images.zip	Archive of 144,487 rendered PNG images
validation.zip	Archive of the 270-diagram manual validation subset
validation/validation_sample.json	Expert-assigned labels for the 270 sampled diagrams
validation/validation_summary.json	Aggregate accuracy statistics over the 270 sampled diagrams
validation/element_type_keys.md	Reference table describing all 57 element type keys
validation/connection_type_keys.md	Reference table describing all 28 connection type keys
validation/puml/	270 PlantUML source files of the validation subset
validation/images/	270 rendered PNG images of the validation subset

The .puml source files and .png images are named by a 40-character SHA-1 hex hash of the source file content (the blob ID). Two patterns produce a numeric suffix appended to the blob ID. Files originating from multi-diagram splitting, where a single source file in the upstream repository contained more than one @startuml/@enduml block, use the pattern {blob_id}_{NN}.puml. The numeric suffix is left-padded with zeros to the width required by the largest index produced from that source file: two digits for source files with 1 to 99 blocks and three digits for 100 or more. The largest split index observed in the dataset is 255. Diagrams using PlantUML's newpage keyword render a single source file to multiple PNG pages under a separate suffix: the first page has no page suffix and each later page takes a fixed three-digit index starting at _001, so a source file yields {blob_id}.png, {blob_id}_001.png, {blob_id}_002.png, and so on. When newpage occurs inside a previously split file, the split index precedes the page index, giving {blob_id}_{MM}.png, {blob_id}_{MM}_001.png, {blob_id}_{MM}_002.png. For example, source blob 5c23ea0c85bdd8c463e1cf136b80ffeecf92c95a was split into diagram blocks, two of which are shown here; newpage rendered each block to three pages, the first page unsuffixed and the next two carrying the _001 and _002 indices:

_01.puml → _01.png, _01_001.png, _01_002.png.

_02.puml → _02.png, _02_001.png, _02_002.png.

The same blob ID keys each per-diagram entry in uml_metadata_enriched.json; a newpage source therefore maps to one metadata record but several rendered images.

Each .puml file is prefixed by three PlantUML comment lines naming the Blob ID, the file's path within the upstream repository (Original Path), and the data source (the constant string “World of Code”). The diagram content preserves the original PlantUML source, except that multi-diagram files were split into individual files and any custom name following @startuml was removed.

The CC BY 4.0 license covers the authors' contributions: the metadata, the validation records, and the curation of the collection. Each .puml source file remains subject to the license of its upstream repository, and each PNG image, as a rendering of its source file, is subject to that same upstream license; this includes the source files and images in the validation subset. The repository and original_path fields (Table 2) identify the source repository, from which the applicable upstream license can be determined, for all but 1424 diagrams (1.0%) whose repository field is null (see Limitations). For these diagrams the upstream license cannot be determined from the dataset metadata; the null repository field identifies all affected records.

**Table 2:** Per-diagram fields in the classifications object.

Field	Type	Description
blob_id	string	SHA-1 content hash (unique identifier)
original_path	string	File path within the source repository
repository	string or null	WoC project identifier; null if unmapped
primary_type	string	Diagram type: class, sequence, activity, state, component, usecase, deployment, object, or timing
secondary_types	array	Additional types for hybrid diagrams; empty for most entries
reasoning	string	LLM classification rationale
content_lines	integer	Non-blank, non-comment line count
elements	object	Element counts keyed by type
elements_total	integer	Total element count
connections	object	Connection counts keyed by type
connections_total	integer	Total connection count
truncated	boolean	True for diagrams whose source exceeded 5000 words and was truncated to 4000 words before classifier input; absent otherwise
extraction_error	string or null	Parser error code; null on success

Every figure and summary statistic describing the dataset is computed from the files listed in Table 1.

### 3.1 Diagram type distribution

Fig. 1 shows the distribution of diagram types in the dataset. The dataset covers 9 of the 14 diagram types defined by UML 2.5: class (51.6%), sequence (24.6%), activity (6.8%), component (5.7%), use case (5.0%), state (3.0%), object (1.7%), deployment (1.4%), and timing (0.1%, 189 diagrams).

A further 982 diagrams (0.7%) carry one or more secondary type labels in their metadata, indicating that they combine elements of more than one UML type within a single file.

### 3.2 Structural metrics

**Fig. 2:**
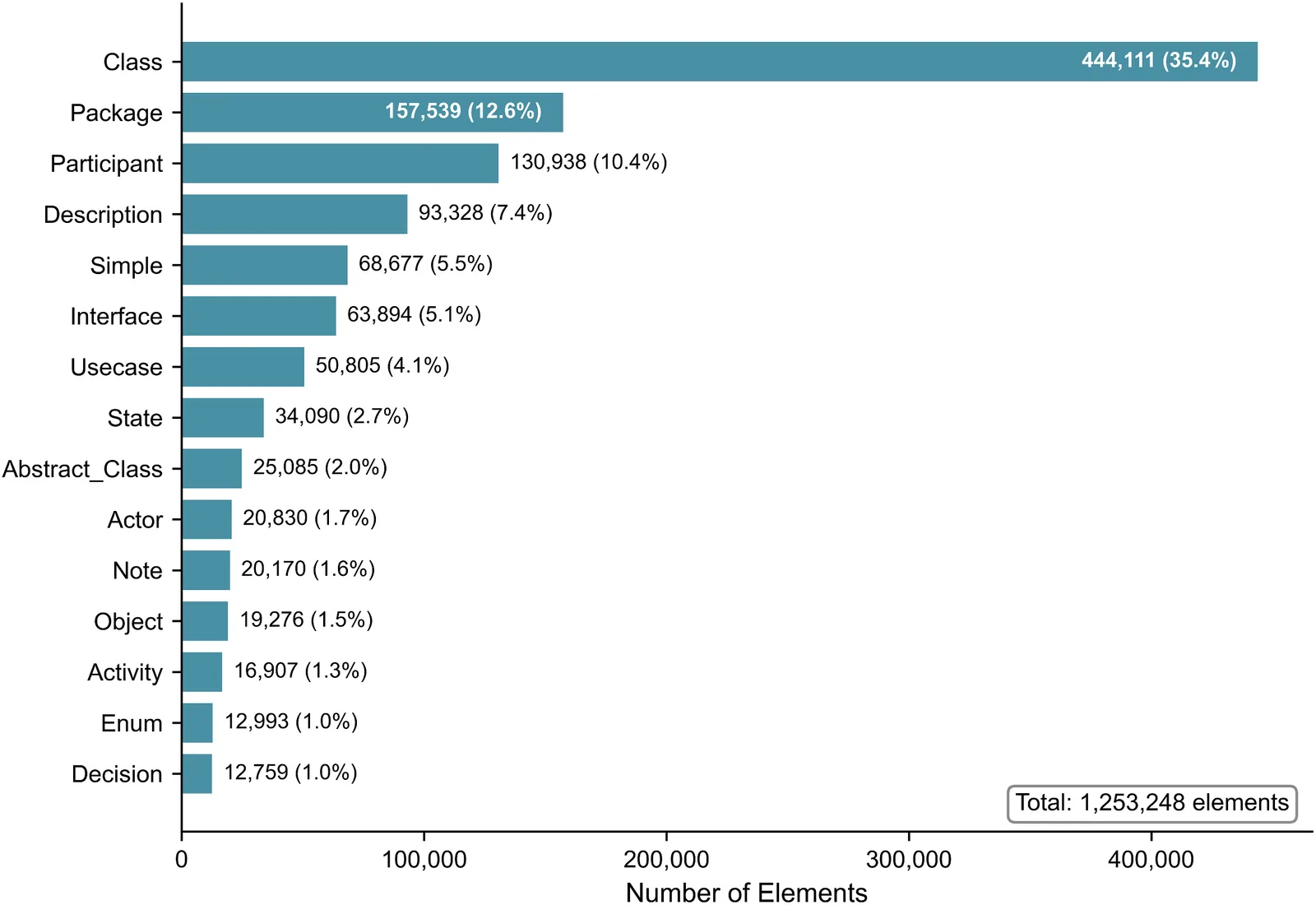
Top 15 element types across all diagrams.

**Fig. 3:**
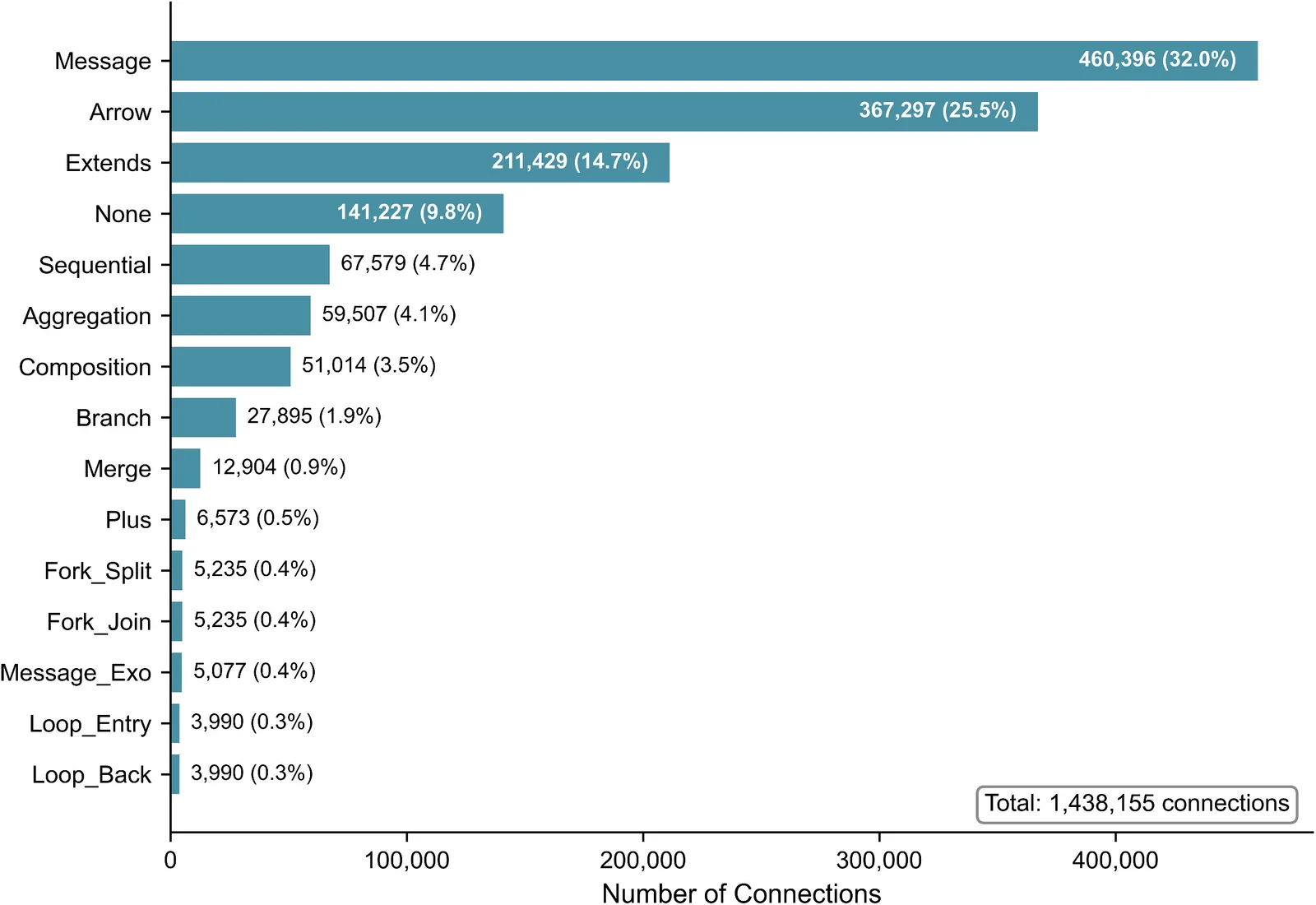
Top 15 connection types across all diagrams.

The uml_metadata_enriched.json file records 1253,248 structural elements of 57 types and 1438,155 connections of 28 types, aggregated across 143,080 of the 143,427 diagrams. Per-diagram element counts have a median of 6 (Q1 3, Q3 10, max 2289). Per-diagram connection counts have a median of 6 (Q1 2, Q3 12, max 10,923). Fig. 2, Fig. 3 show the 15 most frequent element and connection types, respectively.

Type labels are taken from PlantUML's internal parser data model rather than standard UML vocabulary; the full set of element and connection keys is documented in validation/element_type_keys.md and validation/connection_type_keys.md.

Of the 143,427 diagrams, 347 (0.2%) have empty element and connection fields; the extraction_error field in the metadata identifies all affected records (see Limitations).

### 3.3 Metadata schema

The metadata file uml_metadata_enriched.json contains three top-level keys. The metadata key records pipeline parameters (model identifier, timestamp, file counts). The statistics key holds pre-computed aggregate analytics: overall diagram counts; per-type diagram counts (type_distribution); summary statistics and bucketed distributions of per-diagram element, connection, and line counts (e.g., elements_statistics, connections_statistics); dataset-wide element and connection totals per type; and a breakdown of extraction errors. The classifications key contains one entry per diagram, keyed by filename (e.g., “5f95f42b…e89e.puml”). Table 2 lists the fields in each per-diagram entry. Box 1 shows an excerpt of the file.

Box 1. Excerpt of uml_metadata_enriched.json; ellipses mark omitted content.

**Table:** 

**{** **"metadata": {** **"timestamp": “2026-03-05T10:30:58.456467”,** **"model": “claude-haiku-4-5-20251001”,** **…** **},** **"statistics": {** **"type_distribution": {"class": 73958, "sequence": 35233, …},** **"elements_statistics": {"min": 0, "max": 2289, "mean": 8.76, …},** **"connections_statistics": {"min": 0, "max": 10923, "mean": 10.05, …},** **…** **},** **"classifications": {** **"5f95f42b5b392db1c75ab9f5c6eb514ac273e89e.puml": {** **"primary_type": "class",** **"secondary_types": [],** **"reasoning": "This diagram defines a single class …",** **"content_lines": 11,** **"elements": {"class": 1},** **"elements_total": 1,** **"connections": {},** **"connections_total": 0,** **"extraction_error": null,** **"blob_id": "5f95f42b5b392db1c75ab9f5c6eb514ac273e89e",** **"original_path": "/src/main/java/ex42/App.puml",** **"repository": "HaoQNguyen…"** **},** **…** **}** **}**

The repository field uses the WoC project identifier convention: for GitHub-hosted repositories the identifier is {username}_{reponame}, from which the GitHub URL can be constructed as https://github.com/{username}/{reponame}; identifiers of repositories hosted on other forges (10,240 diagrams, 7.1%) begin with the forge domain, as in bitbucket.org_{username}_{reponame}.

### 3.4 Validation records

The validation/ directory holds a 270-diagram subset of the dataset used to assess the accuracy of the LLM-based diagram type classification and the parser-based structural extraction. The expert assignments and aggregate accuracy statistics are recorded in validation_sample.json and validation_summary.json. The reviewed PlantUML source files and rendered PNG images are placed in validation/puml/ and validation/images/. The directory also contains the type-key reference tables element_type_keys.md and connection_type_keys.md, which document the element and connection type vocabularies referenced in these records.

The validation_sample.json file has two top-level keys: metadata (sample size, per-type counts, random seed) and samples (the 270 per-diagram records). Table 3 lists the fields in each per-sample record.

**Table 3:** Per-sample fields in the validation_sample.json samples array.

Field	Type	Description
sample_id	integer	Sequential record identifier (1–270)
filename	string	PlantUML source file name of the sampled diagram
classified_type	string	Diagram type assigned by the LLM-based classifier
content_lines	integer	Non-blank, non-comment line count
elements	object	Element counts produced by the parser-based extraction tool
total_elements	integer	Total element count from the extraction tool
connections	object	Connection counts produced by the parser-based extraction tool
total_connections	integer	Total connection count from the extraction tool
actual_type	string	Diagram type assigned by the expert reviewer
elements_correct	boolean	True if expert and tool element counts agree exactly
actual_elements	object	Element counts as determined by the expert reviewer
actual_elements_count	integer	Total element count as determined by the expert reviewer
connections_correct	boolean	True if expert and tool connection counts agree exactly
actual_connections	object	Connection counts as determined by the expert reviewer
actual_connections_count	integer	Total connection count as determined by the expert reviewer
notes	string or null	Reviewer notes for the sample; null when none recorded

The validation_summary.json file records aggregate statistics across three dimensions: classification, elements, and connections. Each dimension records overall accuracy, per-type accuracy, and the list of mismatched samples. The classification dimension additionally records a confusion matrix; the element and connection dimensions additionally record overcount and undercount tallies, together with mean and median absolute differences.

## 4 Experimental Design, Materials and Methods

**Fig. 4:**
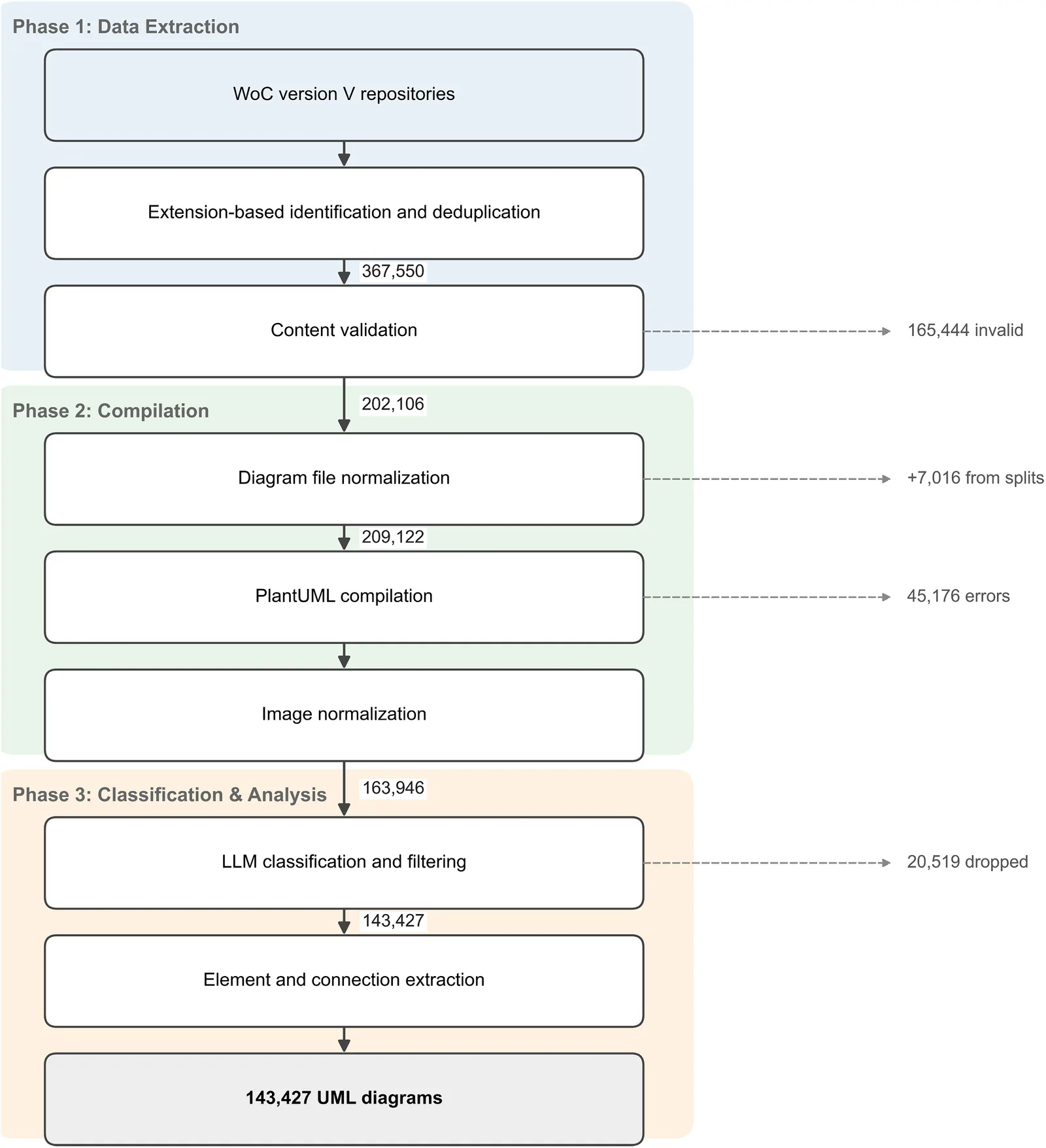
Overview of the data extraction and processing pipeline. Counts on arrows indicate the number of items flowing between steps; dashed arrows show items excluded at each filtering stage and the 7016 diagram files added by multi-diagram splitting.

The dataset was produced by a three-phase pipeline [8] (Fig. 4). Phase 1 identifies PlantUML files in the WoC infrastructure by extension, deduplicates them by content hash, and validates the presence of @startuml/@enduml markers, yielding 202,106 validated source files from 367,550 candidate blobs. Phase 2 splits multi-diagram files into individual units, normalises tag syntax, compiles each source file with PlantUML, and normalizes the rendered images, retaining the 163,946 diagrams that compile successfully. Phase 3 assigns each diagram to one of nine standard UML types using a large language model, drops entries the classifier labelled as non-UML, and extracts per-diagram element and connection counts with a parser-based tool, producing the final collection of 143,427 UML diagrams. Table 4 complements Fig. 4 by tracing the item counts through each pipeline step, including the per-cause exclusion counts and the final PNG count.

**Table 4:** Data flow from candidate files to the final dataset.

Pipeline step	Change	Items after step
Extension-based file identification in WoC basemaps	-	504,451 path entries
Deduplication by blob identifier	−136,901 entries with duplicate blob identifiers	367,550 unique blobs
Content validation	−163,802 lacking @startuml/@enduml; −1642 not retrievable	202,106 validated source files
Multi-diagram splitting	+7016 from 1842 multi-diagram files	209,122 diagram files
PlantUML compilation	−45,176 compilation failures	163,946 compiled diagrams
Non-UML removal (LLM classification)	−20,434 labelled non-uml	143,512 diagrams
Final filtering	−84 Graphviz DOT passthrough; −1 empty diagram	143,427 diagrams
Final dataset: PlantUML source files	-	143,427 .puml files
Final dataset: rendered PNG images	-	144,487 PNG images

Note: Compilation rendered 165,177 PNG images for the 163,946 compiled files (the newpage keyword produces multiple output pages from a single source file); images of diagrams excluded after compilation were removed together with their sources, leaving the final 144,487 images.

### 4.1 Data source

The original data source is open-source git repositories hosted on public platforms (GitHub, GitLab, Bitbucket, and others). The World of Code (WoC) infrastructure [6], maintained by the University of Tennessee, Knoxville, indexes and mirrors these repositories, and was used here as the centralised access mechanism for ecosystem-scale extraction. WoC releases are designated by sequential letters; the dataset is derived from version V, whose snapshot was built from repository updates identified during 1–30 March 2023, with git objects retrieved by mid-May 2023. Version V indexes over 209 million repositories and 16.2 billion unique blobs (file contents).

File identification used the lb2fFull basemap, a 128-shard file collection that maps blob identifiers (SHA-1 hashes of file content) to the file paths under which each blob appears, in the format blob_id;file_path. WoC's content-addressable storage means that one blob identifier denotes one file content regardless of how many repositories or paths contain it.

### 4.2 File identification and content validation

Candidate PlantUML files were identified by a case-insensitive regular expression search across all 128 lb2fFull basemap shards, matching file paths ending with one of six PlantUML-associated extensions: .puml, .pu, .plantuml, .wsd, .iuml, and .uml. The search returned 504,451 file entries. The results were deduplicated by blob identifier, retaining the lexicographically first path per unique SHA-1 hash (via the Unix sort -u command), leaving 367,550 unique candidate blobs.

The content of each candidate blob was then retrieved from WoC storage and checked for the presence of PlantUML boundary markers. A blob was accepted only if it contained both an @startuml and an @enduml marker, matched as a case-insensitive substring. Of the 367,550 candidates, 202,106 (55.0%) passed this validation, 163,802 (44.6%) lacked the required markers, and 1642 (0.4%) could not be retrieved from WoC storage.

Per-extension validation rates are shown in Table 5. All six extensions were retained in the extraction; the extension with the lowest validation rate (.uml) still contributed 7186 valid blobs to the candidate set.

**Table 5:** PlantUML content validation rate by file extension.

Extension	Valid	Invalid	Total	Valid Rate (%)
.plantuml	21,980	746	22,726	96.7
.puml	146,851	25,766	172,617	85.1
.pu	10,004	2472	12,476	80.2
.wsd	14,862	8709	23,571	63.1
.iuml	1223	1109	2332	52.4
.uml	7186	124,998	132,184	5.4

Note: Excludes 2 blobs with no recognised extension (both invalid) and 1642 blobs that could not be retrieved from WoC storage.

The extension-based approach was chosen because a content-based alternative (scanning all 16.2 billion blobs in WoC for @startuml markers) would be computationally prohibitive. The six targeted extensions match those identified by Romeo et al. [5]. The approach does not capture PlantUML content stored in files with non-standard extensions or embedded within other document formats such as Markdown or AsciiDoc.

### 4.3 Diagram file normalization

Two normalization steps were applied to the 202,106 validated source files before compilation: multi-diagram splitting and tag normalization.

PlantUML allows multiple diagrams to be defined within a single file using successive @startuml/@enduml blocks. Of the 202,106 validated files, 1842 (0.9%) contained two or more diagram blocks. A splitting algorithm detected all @start{type}/@end{type} pairs (including non-UML types such as @startditaa and @startsalt) using case-insensitive regular expression matching, and extracted each block into a separate file named {blob_id}_01.puml, {blob_id}_02.puml, and so on, with the metadata header copied to every resulting file to preserve provenance. Single-diagram files were left unchanged. This step increased the file count from 202,106 to 209,122, yielding 7016 additional files.

A tag normalization step then addressed two issues that would otherwise interfere with compilation. PlantUML's @startuml directive accepts an optional name argument (via braces, parentheses, brackets, or a space-separated token) that overrides the output filename; retaining custom names would produce PNG files whose names no longer match their source files, breaking the metadata linkage. Additionally, commented-out diagram directives (e.g., '@startuml) can cause the compiler to generate spurious output images. Two in-place modifications were applied: stripping name arguments from @start{type} directives, and removing commented-out @start/@end lines. Of the 209,122 files, 35,612 (17.0%) required at least one of these modifications. The total file count was unaffected by this step.

### 4.4 Compilation

The 209,122 preprocessed .puml files were compiled with PlantUML version 1.2025.9 at 300 DPI with the rendering size cap set to 16,384 pixels. The compiler was invoked with the –no-error-image flag, which suppresses the placeholder error image otherwise generated for files that fail to compile; with the flag enabled, the presence of a PNG file serves as a direct indicator of successful compilation.

Of the 209,122 input files, 163,946 (78.4%) compiled successfully, producing 165,177 PNG images because PlantUML's newpage keyword splits a single diagram into multiple output pages; 45,176 (21.6%) produced compilation errors. The compilation step also acted as a syntactic validation layer; only the 163,946 successfully compiled diagrams proceeded to diagram type classification.

### 4.5 Image normalization

A post-processing pass applied two transformations to the compilation output. Each image with width or height below 512 pixels was placed at the centre of a canvas whose width and height equalled the corresponding image dimension or 512 pixels, whichever was larger. The pad colour was sampled from the top-left pixel of the original image so that diagrams with custom background themes do not appear with a mismatched white frame. All images were then converted to RGB colour mode, replacing the palette, RGB, and RGBA encodings that PlantUML's renderer produces depending on diagram content. The PNG filename was preserved at each step to maintain the linkage to source files and metadata. No data augmentation (rotation, flipping, scaling, cropping, or colour adjustment) was applied at any stage.

The minimum 512-pixel resolution and uniform RGB colour mode align the image component of the dataset with the Data in Brief machine-learning image-dataset policy (11 June 2025).

### 4.6 Diagram type classification

PlantUML uses a single @startuml keyword for all nine standard UML diagram types and does not record the diagram type explicitly in the source file. To assign a type label to each of the 163,946 compiled diagrams, Claude Haiku 4.5 (model identifier claude-haiku-4-5-20251001) was used via the Anthropic Message Batches API. Each diagram's preprocessed PlantUML source was submitted with a structured prompt instructing the model to classify by semantic meaning (content and intent) rather than syntax alone. The prompt was sent as a single user message with no system prompt; the model was queried with a maximum of 256 output tokens, and the sampling parameters (temperature and top-p) were left at their Anthropic API defaults (temperature 1.0). Requests were submitted in batches of up to 100,000. The full prompt is shown in Box 2.

Box 2. The diagram-type classification prompt.

**Table:** 

**Classify this PlantUML diagram by its semantic meaning, not just syntax.** **UML TYPES:** **- sequence: interactions between participants over time (participant, ->, activate, alt, loop, group)** **- class: type definitions with attributes and/or methods, and their relationships. Keywords: class, interface, enum, struct, abstract, entity, extends, implements. Includes diagrams with only attributes (no methods), domain models, and data structure definitions.** **- activity: workflow/process flow (start, stop, :action;, if/then/else, fork, partition)** - state: state machine transitions ([*], state, –>) **- usecase: actors interacting with use cases ((usecase), :actor:, actor keyword)** **- component: system components and their interfaces ([component], interface, package, rectangle)** **- deployment: physical infrastructure (node, artifact, device, cloud, database as infrastructure)** **- object: concrete object instances showing runtime values (object keyword, e.g., object "o1:Order" with specific field values)** **- timing: timing constraints and signals (@time, robust, concise)** **NON-UML TYPES:** **- non-uml: anything that is NOT a standard UML diagram. Includes:** **- PlantUML-specific formats: @startmindmap, @startgantt, @startwbs, @startjson, @startyaml, @startsalt, @startditaa, @startnwdiag, @startdot** **- ERD / database schemas: diagrams that specifically use crow's foot notation (||–o{,}o–|{, ||–|{) AND database column types (INT, VARCHAR, TEXT, BOOL, PK, FK). Both indicators must be present to classify as ERD.** **- Graphviz DOT passthrough: diagrams using raw `digraph` or `graph` DOT syntax inside @startuml (PlantUML forwards these to Graphviz without building a UML model)** **- Other non-UML notations drawn using PlantUML syntax** **- Empty diagrams, macro/sprite-only files, or anything not recognizable as a diagram type** **OUTPUT FORMAT (JSON only, no other text):** **{** **"primary_type": "<type>",** **"secondary_types": [],** **"reasoning": "<1-sentence explanation>"** **}** **RULES:** **1. Most diagrams have exactly ONE type. Return it as primary_type with empty secondary_types.** **2. Only add secondary_types when the diagram genuinely combines multiple UML diagram types in a single file (e.g., a sequence diagram that also defines class relationships). This is rare.** **3. If the diagram uses @startmindmap, @startgantt, @startwbs, @startjson, @startyaml, @startsalt, @startditaa, @startnwdiag, or @startdot, return primary_type: "non-uml".** 4. ERD detection: only classify as non-uml ERD when the diagram has BOTH crow's foot notation (||–o{,}o–|{) AND database-specific column types (INT, VARCHAR, TEXT, PK, FK). Using "entity" or "class" keyword alone does not make it an ERD — diagrams with attributes, compositions (*–), or inheritance are class diagrams. **5. If the diagram body starts with `digraph` or `graph {` (raw Graphviz DOT syntax inside @startuml), return primary_type: "non-uml".** **6. If no recognizable patterns at all, return primary_type: "non-uml".** **PlantUML DIAGRAM:** **```** **__CONTENT__** **```**

Note: The __CONTENT__ placeholder is replaced with each diagram's preprocessed PlantUML source before submission; the same prompt is available in the archived pipeline code [8].

Before classification, each diagram was passed through a lightweight preprocessing pipeline that reduced token consumption while preserving signals relevant to type identification. The pipeline removed the metadata headers (blob ID, original path, and source comments), sprite definitions (both hex/raster and SVG inline sprites), preprocessor procedure and function bodies, hyperlink syntax, and documentation blocks (headers, footers, legends, and captions). Multi-line notes were collapsed to single-line markers and excessive blank lines were reduced. The pipeline preserved elements that carry strong type signals: human-authored comments (which often describe diagram purpose), styling directives (skinparam, hide, show), standard library includes (!include), macro definitions (!define), and title lines. Diagrams whose raw source exceeded 5000 whitespace-delimited words were truncated to the first 4000 words of the preprocessed text before classification; the truncated field identifies the affected records.

For each diagram, the classifier produced the primary_type, secondary_types, and reasoning fields documented in Table 2. A secondary type was recorded only when a diagram genuinely combines more than one UML type in a single file, which is rare. Diagrams not matching any of the nine standard UML types received the non-uml label.

Of the 163,946 classified diagrams, 20,434 (12.5%) were assigned the non-uml label. This category comprises predominantly sprite and icon library definitions, PlantUML-specific non-UML formats (@startmindmap, @startgantt, @startwbs, @startjson, @startyaml, @startsalt, @startditaa, @startnwdiag, @startdot), ERD and database schemas using crow's foot notation, and empty or unrecognisable files. All 20,434 entries were removed, reducing the dataset to 143,512 diagrams. Two additional filters were applied. First, 84 diagrams were identified as Graphviz DOT passthrough (raw digraph/graph syntax wrapped in @startuml that PlantUML forwards to Graphviz without constructing a UML model). These were detected by the parser-based extraction tool described in the next subsection, which reports the error code unsupported_type:PSystemDot for such files. Second, one diagram with zero content lines was removed. The resulting dataset contains 143,427 UML diagrams.

### 4.7 Element and connection extraction

To quantify diagram structure, a parser-based extraction tool was developed that counts typed elements (nodes) and connections (edges) for each diagram. The tool reuses PlantUML's own parsing pipeline rather than regular expression matching, so that element and connection counts reflect the same interpretation of the source that the compiler applies when rendering, including macro expansion, !include resolution, conditional compilation, and alias resolution.

The tool is implemented as a Java class, DiagramStatsExtractor, embedded within a fork of PlantUML version 1.2025.9 [9]. For each input file, it invokes PlantUML's full parsing pipeline to produce a fully resolved diagram object, then inspects this object to extract per-type element and connection counts. No modifications were made to PlantUML's parsing logic, command definitions, or diagram construction code.

Three extraction strategies (entity-link, sequence, and activity) are dispatched based on the diagram's internal representation. The entity-link strategy handles class, object, component, deployment, use case, and state diagrams, which share a common internal representation with separate entity and link collections. Elements are read from the entity collection and typed by their internal label; connections are read from the link collection, with each connection typed by the more semantically specific end when a link carries different decorations on each end (priority order: extends, composition, aggregation, arrow, none).

The sequence strategy handles sequence diagrams. Declared participants are counted as elements by their type. Messages between participants are counted as connections, with messages crossing the diagram boundary recorded separately. Combined fragments (alt, opt, loop), notes, dividers, and references are not counted, as they are behavioural annotations rather than graph structure.

The activity strategy handles activity diagrams by traversing PlantUML's tree-structured intermediate representation. Nodes are counted as elements with labels derived from PlantUML's internal instruction types, and control flow edges between nodes are counted as connections. These edges represent the abstract control flow graph rather than visual arrows in the rendered diagram.

Two characteristics of PlantUML's internal representation affect the resulting counts. First, in entity-link diagrams, PlantUML injects synthetic links into its link collection for rendering purposes (note-attachment links that anchor notes to entities, and layout links generated for internal arrangement); these carry no distinguishing marker, so the tool cannot separate them from user-declared undecorated associations, and both appear as none connections. Second, in activity diagrams, the tool counts convergence edges for compound instructions (if/switch/fork blocks) unconditionally, even when all branches terminate before reaching the merge point. In both cases the tool counts connections that are not present in the source, so the connection totals can be over-estimates but never under-estimates.

Of the 143,427 diagrams in the dataset, 143,080 were successfully processed by the extraction tool; the remaining 347 are reported with an extraction_error code in the metadata.

### 4.8 Validation

A stratified random sample of 270 diagrams (30 per classifier-assigned type across all 9 types) was selected with a fixed random seed (42) for reproducibility. A single domain expert (the first author) reviewed each diagram by reading the PlantUML source file. The review followed a two-layered design. In Layer 1, the reviewer independently determined the diagram type and counted total elements and connections without consulting the tool's output, eliminating confirmation bias on aggregate totals. In Layer 2, the reviewer examined the tool's per-type breakdown against the 57 element type keys and 28 connection type keys, verifying or correcting individual counts. The validation is source-based rather than visual: the extraction tool operates on PlantUML's parsed intermediate representation, and activity diagram connections represent control-flow graph edges that have no one-to-one visual counterparts. The complete validation records are released in the validation/ directory of the dataset.

**Fig. 5:**
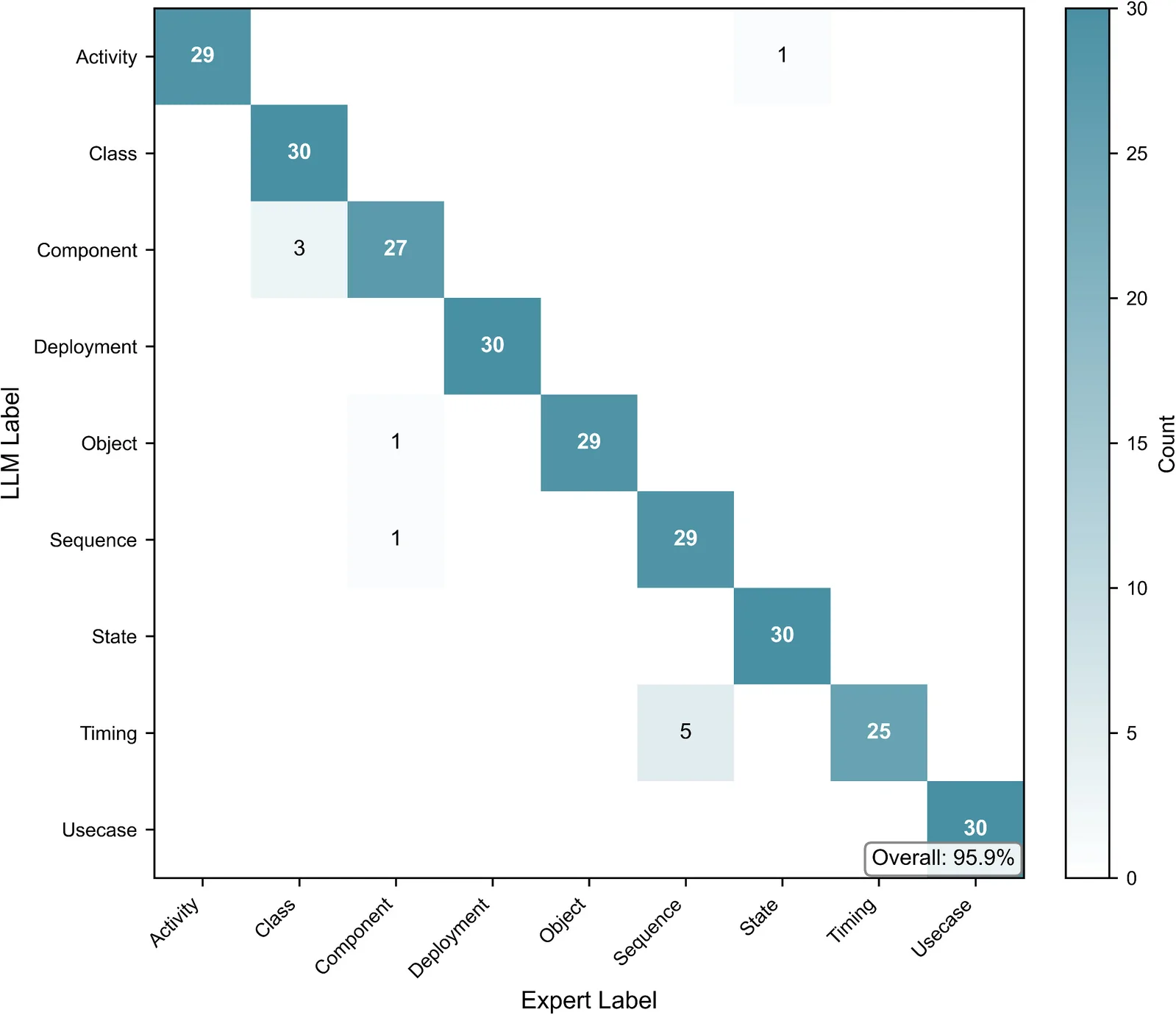
Confusion matrix for diagram type classification on the 270-diagram validation sample. Rows represent the LLM-assigned type; columns represent the expert-assigned type. Off-diagonal entries indicate misclassifications.

The expert-assigned type matched the LLM-assigned type for 259 of the 270 sampled diagrams (95.9%; 95% Wilson score confidence interval [CI]: 92.9–97.7%). Four diagram types (class, deployment, state, and use case) had no misclassifications (30/30 each). Fig. 5 shows the confusion matrix. The pooled agreement and its confidence interval are results for the balanced validation sample rather than an estimate of dataset-wide classification accuracy, because the sample contains 30 diagrams per type whereas the dataset's type distribution is strongly unequal (Fig. 1).

**Fig. 6:**
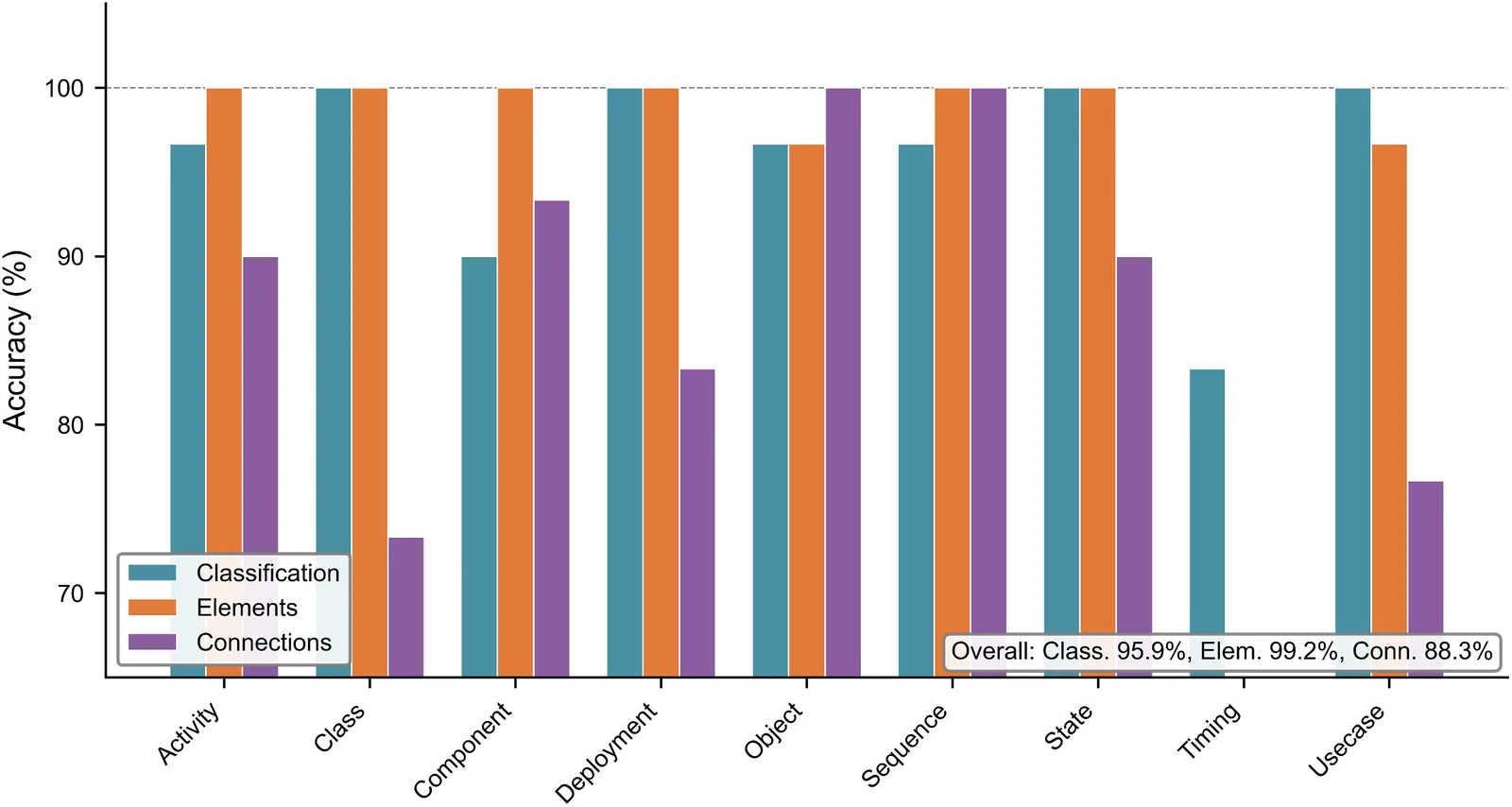
Per-type validation results for classification, elements, and connections. Classification bars show agreement between the LLM-assigned and expert-assigned type; element and connection bars count a diagram as an exact match when both the per-type breakdown and the aggregate total agree with the expert review. Element and connection bars are absent for timing diagrams due to extraction limitations.

Element and connection extraction accuracy was evaluated on 240 diagrams; the 30 timing diagrams were excluded because 178 of the 189 timing diagrams in the full dataset (94.2%) have empty extraction fields. Element extraction achieved a 99.2% exact match rate (238/240; 95% CI: 97.0–99.8%); both mismatches are cases where the tool returned zero elements for a diagram that contains them, one in an object diagram split by newpage directives and one in a use case diagram. Connection extraction achieved an 88.3% exact match rate (212/240; 95% CI: 83.7–91.8%), with 27 overcounts and 1 undercount, and a median overcount of 2 connections among affected diagrams. These mismatches are dominated by the two extraction biases described in the previous subsection. Fig. 6 presents the per-type breakdown of classification agreement and element and connection exact match rates.

## Limitations

The dataset is derived from World of Code version V, whose snapshot was built from repository updates collected during March–May 2023, and does not include diagrams added or modified after that period. PlantUML files are identified by six file extensions (.puml, .pu, .plantuml, .wsd, .iuml, .uml); PlantUML content stored under non-standard extensions or embedded within Markdown, AsciiDoc, or build-tool configuration files is not captured. Deduplication uses the SHA-1 content hash, so near-duplicate diagrams differing by whitespace or minor edits are retained as separate entries. All diagrams were compiled with PlantUML version 1.2025.9, and other versions may parse or render syntax differently. For 347 diagrams (0.2%), the parser-based extraction tool produced empty element and connection fields, predominantly for timing diagrams (178) and diagrams using the newpage keyword (167); these diagrams should be excluded from automated structural analyses. Connection counts carry two extraction biases that inflate them (synthetic links and unconditional convergence edges), described in the Element and Connection Extraction subsection of Methods. The manual validation relied on a single rater (the first author); inter-rater agreement could therefore not be assessed. For 1424 diagrams (1.0%), the WoC blob-to-project mapping did not provide a source repository identifier.

## CRediT Author Statement

**Volodymyr Polishchuk**: Conceptualization, Methodology, Software, Validation, Formal Analysis, Investigation, Data Curation, Visualization, Writing - Original Draft. **Vladyslava Skidan**: Supervision, Writing - Review & Editing.

## Ethics Statement

The authors have read and follow the ethical requirements for publication in Data in Brief and confirm that the current work does not involve human subjects, animal experiments, or any data collected from social media platforms. The dataset consists of PlantUML source files extracted from publicly available open-source git repositories. No dedicated scan for personally identifiable information or inadvertently committed secrets (such as credentials or API keys) was performed; the files are redistributed as they already appear in these public repositories, which World of Code indexes and mirrors. Per-file provenance (repository identifier, original path, and SHA-1 content hash) is recorded so that each file can be traced to its source repository and its upstream license; for 1424 entries (1.0%) the repository identifier is unavailable (see Limitations). The CC BY 4.0 license applies to the authors' contributions (the metadata, validation records, and the curation of the collection); the source files and their rendered images remain subject to their respective upstream repository licenses.

## Data Availability

ZenodoUML-in-the-Wild: A Dataset of UML Diagrams in PlantUML Notation from Open-Source Repositories (Original data)

## References

[1] Bates A., Vavricka R., Carleton S., Shao R., Pan C. (2025). Unified modeling language code generation from diagram images using multimodal large language models. Mach. Learn. Appl..

[2] Conrardy A., Cabot J. (2024). Proc. LLM4MDE Workshop at STAF 2024.

[3] Hebig R., Ho-Quang T., Chaudron M.R.V., Robles G., Fernández M.A. (2016). Proc. ACM/IEEE 19th Int. Conf. Model Driven Engineering Languages and Systems (MODELS).

[4] Robles G., Ho-Quang T., Hebig R., Chaudron M.R.V., Fernández M.A. (2017). Proc. 14th Int. Conf. Mining Software Repositories (MSR).

[5] Romeo J., Raglianti M., Nagy C., Lanza M. (2025). Proc. 47th Int. Conf. Software Engineering (ICSE).

[6] Ma Y., Dey T., Bogart C., Amreen S., Valiev M., Tutko A., Kennard D., Zaretzki R.L., Mockus A. (2021). World of code: enabling a research workflow for mining and analyzing the universe of open source VCS data. Empir. Softw. Eng..

[7] Polishchuk V., Skidan V. (2026).

[8] Polishchuk V. (2026).

[9] Polishchuk V. (2026).

